# Evidence for loss of nepotism in the evolution of permanent sociality

**DOI:** 10.1038/srep13284

**Published:** 2015-09-03

**Authors:** Reut Berger-Tal, Yael Lubin, Virginia Settepani, Marija Majer, Trine Bilde, Cristina Tuni

**Affiliations:** 1 Mitrani Department of Desert Ecology, Blaustein Institutes for Desert Research, Ben-Gurion University of the Negev, Sede Boqer Campus, Midreshet Ben-Gurion, 84990, Israel; 2 Aarhus University, Department of Bioscience, Ny Munkegade 116, DK-8000 Aarhus C, Denmark; 3 BioCircuits Institute University of California, San Diego 9500 Gilman Drive, Mail Code 0328 La Jolla, CA 92093-0328, USA; 4 Ludwig Maximilians University Munich, Department Biology II, Großhaderner Str. 2, 82152 Planegg–Martinsried, Germany

## Abstract

Kin selected benefits of cooperation result in pronounced kin discrimination and nepotism in many social species and favour the evolution of sociality. However, low variability in relatedness among group members, infrequent competitive interactions with non-relatives, and direct benefits of cooperation may relax selection for nepotism. We tested this prediction in a permanently social spider, *Stegodyphus dumicola* that appears to fulfil these conditions. Sociality is a derived trait, and kin discrimination exists in sub-social closely related congeners and is likely a selective force in the sub-social route to permanent sociality in spiders. We examined whether social spiders show nepotism in cooperative feeding when genetic relatedness among group members was experimentally varied. We found no effect of relatedness on feeding efficiency, growth rate or participation in feeding events. Previous studies on sub-social species showed benefits of communal feeding with kin, indicating nepotistic cooperation. The lack of evidence for nepotism in the social species suggests that kin discrimination has been lost or is irrelevant in communal feeding. Our results are consistent with the hypothesis that the role of nepotism is diminished when cooperation evolves in certain genetic and ecological contexts, e.g. when intra-group genetic relatedness is homogeneous and encounters with competitors are rare.

Kin selection theory predicts that individuals gain inclusive fitness benefits from cooperating with relatives, because the costs of helping is outweighed by the increased fitness of individuals with whom they share genes identical by decent^1^,^2^. Coined as Hamilton’s rule, it follows that inclusive fitness gained through helping behaviour will generally increase with the relatedness coefficient between helper and receiver^1^,^2^. This should select for kin discrimination, as directing help towards relatives requires the ability to distinguish kin from non-kin, based on either environmentally or genetically determined cues^3^,^4^. Indeed, there is wide evidence for kin discrimination in social animals, emphasizing the significance of nepotism, i.e. preferential cooperation with kin, in social evolution^5^,^6^. Given that recognition systems are costly to maintain, kin discrimination is expected to be most pronounced when there are significant inclusive fitness benefits of nepotism^5^. If these conditions are relaxed, or cooperation occurs as a by-product to otherwise selfish actions, selection for kin discrimination may be diminished and recognition systems lost. This could explain the apparent absence of kin discrimination and nepotism in a range of cooperative species^5^,^7^,^8^,^9^,^10^. There are several contexts under which we might expect relaxed selection for nepotism in cooperating groups^11^,^12^: 1) when benefits of cooperative traits that directly increase group productivity^7^,^13^,^14^, e.g. group foraging and task differentiation^15^,^16^, override benefits of nepotism, 2) if there is low variation in genetic relatedness among cooperating individuals, inclusive fitness benefits of cooperation will be retained while the maintenance of a recognition system may be unnecessary^5^,^17^,^18^,^19^; and 3) in situations where there is little competitive interaction with non-relatives, the cost of maintaining a recognition system may outweigh the benefits^10^,^20^.

While the benefits of kin discrimination in directing help towards relatives seem unequivocal in the evolution of cooperation, the role of nepotism in maintaining and structuring social groups once cooperation has evolved remains enigmatic. Using permanently social spiders as our study system, we tested the prediction that nepotism can be lost under conditions where kin recognition may no longer be beneficial. Social spiders form colonies consisting of highly related individuals, owing to within-colony breeding and lack of pre-mating dispersal^21^,^22^. This results in highly genetically homogeneous individuals within groups^23^ and limited gene flow among colonies^23^,^24^,^25^. Colony members cooperate in brood care, nest building, web maintenance, feeding and prey capture^21^. Sub-social species in the same genus are characterized by extended maternal care and a transient cooperative juvenile stage, followed by pre-mating dispersal and a solitary adult life^21^,^22^. Sociality in spiders is suggested to have evolved via the sub-social route^26^, where ecological constraints on dispersal in sub-social progenitors may have favoured juvenile philopatry and elimination of pre-mating dispersal, leading to the formation of cooperatively foraging groups^27^,^28^. Cooperative foraging, where spiders jointly subdue and feed on a captured prey may create the potential for within-group competition and, due to spiders feeding mode, it is open to exploitation. This is because some individuals may invest in prey capture and costly digestive enzymes that serve to feed by liquefying the prey content, while others may feed with little prior investment^29^, resulting in the depletion of the common resource. Sub-social species may solve this problem, also known as ‘the tragedy of the commons’, and reduce the disadvantages of kin competition through kin-biased cooperation since the costs of cheating relatives through selfish acts are high^30^,^31^. Indeed, net benefits of foraging with kin revealed by improved feeding efficiency and growth rates of groups with higher genetic relatedness, are documented in two sub-social species of the genus *Stegodyphus*, suggesting that kin discrimination is an important factor in the evolution of group living in these spiders^32^,^33^. In contrast, benefits of kin discrimination once permanent sociality has evolved are less clear, as high relatedness and genetic similarity among group members^18^, infrequent encounters with non-kin owing to a sedentary life-style^21^, and direct benefits of cooperation^7^,^13^, may lead to relaxed selection for kin discrimination.

We investigated whether kin discrimination exists in cooperative foraging in the permanently social spider *Stegodyphus dumicola* (Eresidae). To examine evidence for nepotism, we manipulated relatedness levels among groups of interacting spiders engaged in cooperative feeding in three distinct experiments. In each experiment we used geographical distances between colonies as a proxy for genetic similarity^23^,^26^. Spiders that originated from the same colony were considered siblings, since colonies are established by single sib-mated females^34^. In the first experiment we established three treatments: groups of siblings originating from the same colony, groups of non-siblings from different colonies within the same population, and groups of non-siblings from colonies originating from different populations. In a second experiment we used double-mated females to create genetic variability in the offspring^35^. We established treatment groups consisting of offspring broods of similar or mixed genetic composition^36^,^37^. Females were mated with two of their brothers, with two males from different colonies within the same population, or with two males from different colonies originating from different populations. In an additional two groups, females were mated with one brother and one male from within either the same or a different population. In both experiments we assessed spider growth rate over a period of 7–9 weeks, the number of spiders participating in communal feeding and the proportion of prey mass extracted (feeding efficiency) in cooperative feeding events^33^. In a third experiment, we compared sib groups (same colony) and mixed groups (multiple colonies) of individually marked spiders to examine whether kinship affected individual participation in feeding events.

If spiders show nepotism in communal feeding, we should detect higher growth rate and feeding efficiency in groups with higher relatedness among individuals as documented in sub-social species^32^,^33^, whereas the absence of these effects of kinship would suggest a diminished role of nepotism in the permanently social *S. dumicola*.

## Results

### Experiment 1

**Figure 1:**
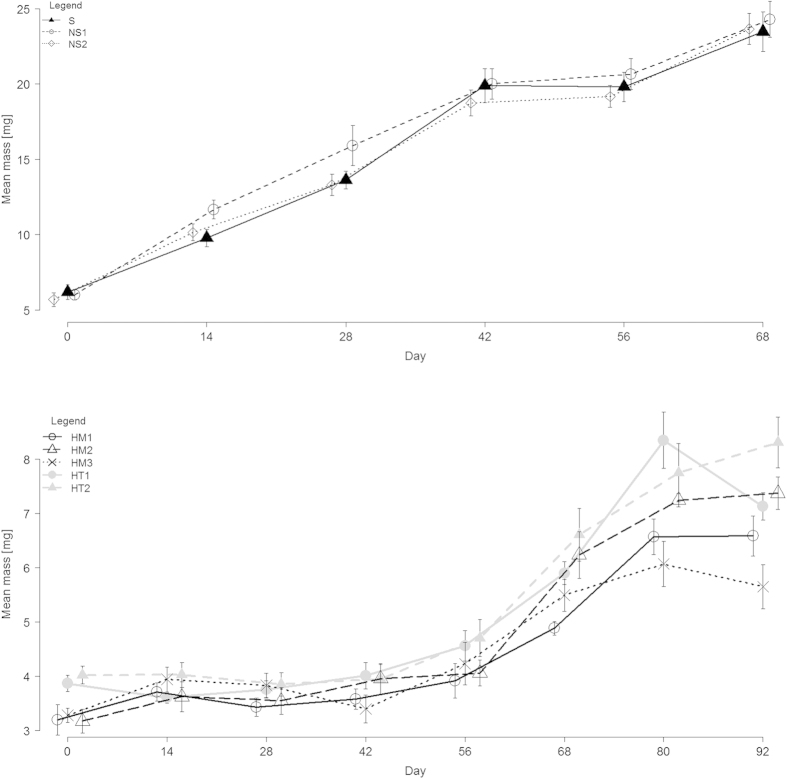
Mean mass of spider groups (mg ± SE) per treatment over the experiment duration (days) in: **(A)** Experiment 1, sibs S (n = 19); non-sibs from same NS_1_ (n = 17) and different populations NS_2_ (n = 17); **(B)** Experiment 2, ‘homogeneous’ broods fathered by two sibs HM_1_ (n = 13), two non-sibs from the same population HM_2_ (n = 8) and from a different population HM_3_ (n = 4); and ‘heterogeneous’ broods fathered by a sib and a non-sib from same HT_1_ (n = 36) and different population HT_2_ (n = 28). In both experiments there was no difference among treatments in mass increase.

**Figure 2:**
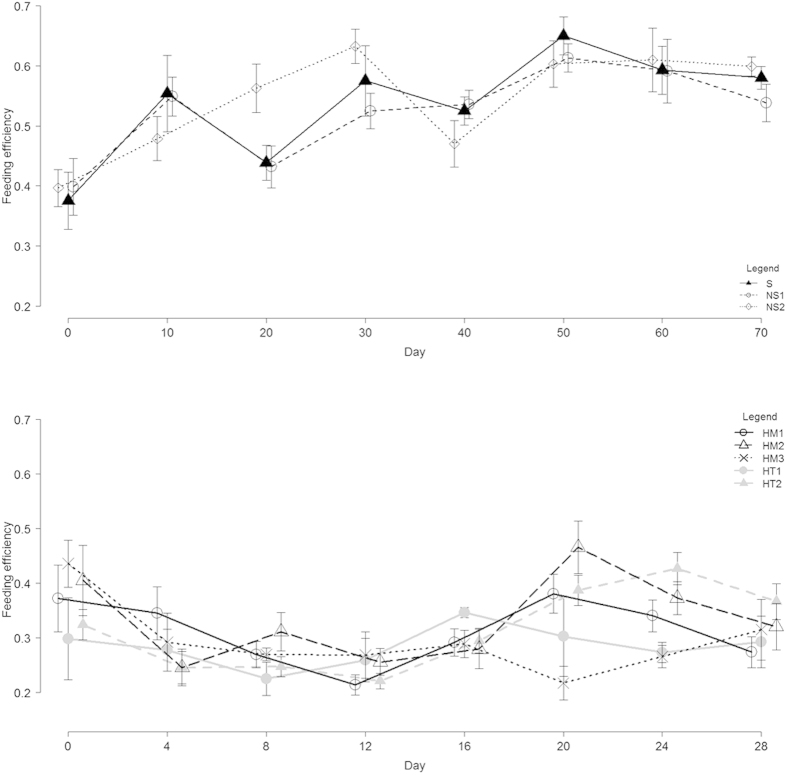
Feeding efficiency (proportion of mass extracted from prey) per treatment over the experiment duration (days) in: **(A)** Experiment 1, sibs S (n = 19); non-sibs from same NS_1_ (n = 17) and different populations NS_2_ (n = 17); **(B)** Experiment 2, ‘homogeneous’ broods fathered by two sibs HM_1_ (n = 13), two non-sibs from the same population HM_2_ (n = 8) and from a different population HM_3_ (n = 4); and ‘heterogeneous’ broods fathered by a sib and a non-sib from same HT_1_ (n = 36) and different population HT_2_ (n = 28). In both experiments there was no difference among treatments in prey mass loss.

**Figure 3:**
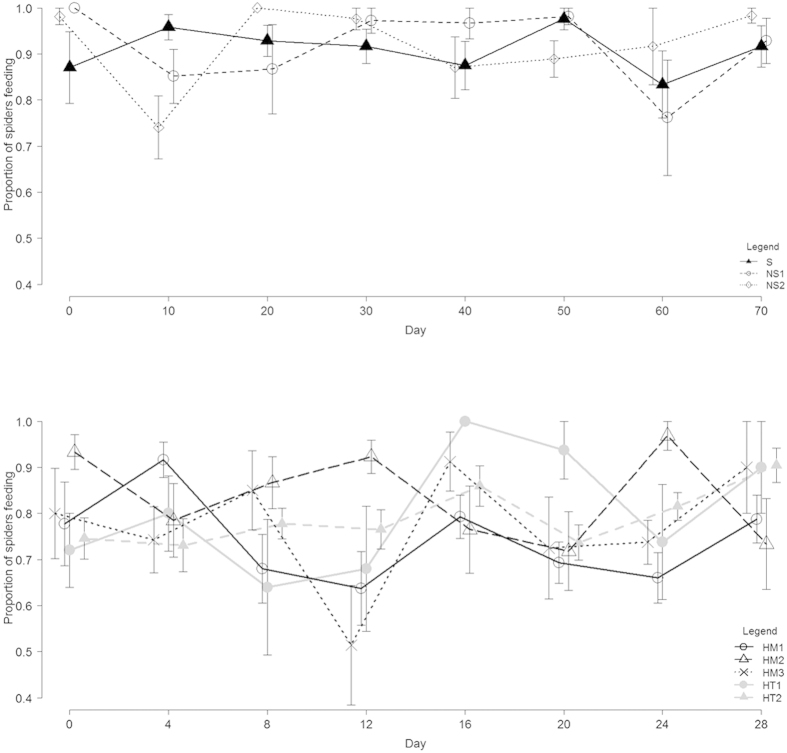
Maximum proportion of spiders feeding per treatment over the experiment duration (days) in: **(A)** Experiment 1, sibs S (n = 19); non-sibs from same NS_1_ (n = 17) and different populations NS_2_ (n = 17); **(B)** Experiment 2, ‘homogeneous’ broods fathered by two sibs HM_1_ (n = 13), two non-sibs from the same population HM_2_ (n = 8) and from a different population HM_3_ (n = 4); and ‘heterogeneous’ broods fathered by a sib and a non-sib from same HT_1_ (n = 36) and different population HT_2_ (n = 28). In both experiments there was no difference among treatments in proportion feeding.

We detected no differential growth rate among different treatment groups over the course of the experiment (Fig. 1A). If nepotistic cooperation resulted in differential growth rate over time, this would be revealed in a significant interaction term^33^, however mixed-model analyses of variance strongly indicated that groups show similar growth rates (Treatment x Time interaction F = 0.46, df = 10, n = 256, p = 0.91). While growth rate changed over time (Time (ordinal effect) F = 240.01, df = 5, p < 0.0001), no difference among treatment groups was detected (Treatment (fixed effect) F = 0.67, df = 2, p = 0.51). Feeding efficiency (proportion of mass extracted from prey) varied over time with no treatment effects (Fig. 2A, ANCOVA, Treatment x Time interaction F = 0.27, df = 2, n = 181, p = 0.76; Time F = 46.19, df = 1, p < 0.0001; Treatment F = 0.09, df = 2, p = 0.91). No difference in the maximum proportion of spiders feeding communally was detected among treatment groups (Fig. 3A, GLM binomial error, Treatment x Time interaction χ^2^ = 1.89, df = 2, n = 181, p = 0.38; Treatment χ^2^ = 0.23, df = 2, p = 0.88; Time χ^2^ = 0.0056, df = 1, p = 0.94). Finally, latency to reach the maximum feeding group size observed in a feeding event also did not differ significantly among groups (mean ± SE (minutes), 1^st^ measurement, S 22.4 ± 5.1, NS_1_ 19.3 ± 3.9, NS_2,_ 24.4 ± 4.4; last measurement, S 22.8 ± 6.1, NS_1_ 22.0 ± 7.2, NS_2,_ 15.0 ± 7.2; GLM exponential error, Treatment x Time interaction χ^2^ = 0.27, df = 2, n = 181, p = 0.87; Treatment χ^2^ = 0.25, df = 2, p = 0.88; Time χ^2^ = 0.72, df = 1, p = 0.39).

### Experiment 2

We detected no differential growth rate of the different treatment groups over the course of time (Fig. 1B, mixed-model ANOVA, Treatment x Time interaction F = 0.60, df = 28, n = 586, p = 0.94). Growth rate changed over time (Time (ordinal effect) F = 36.63, df = 7, p < 0.0001), and a marginally significant effect indicated that treatment groups varied slightly in growth rate (Treatment F = 2.41, df = 4, p = 0.048). Feeding efficiency (arcsine-transformed proportion of mass extracted from prey) varied over time with no consistent treatment effect (Fig. 2B, ANCOVA, Treatment x Time interaction F = 4.03, df = 4, n = 563, p = 0.003; Time F = 1.76, df = 1, p = 0.18; Treatment F = 1.04, df = 4, p = 0.38). The maximum proportion of spiders feeding communally varied among treatment groups over the course of the experiment (Fig. 3B, GLM binomial error, Treatment x Time interaction χ^2^ = 15.21, df = 1, n = 456, p = 0.004; Treatment χ^2^ = 17.1, df = 4, p = 0.0018; Time χ^2^ = 3.57, df = 1, p = 0.058). Contrast analysis showed that these differences were not attributed to more related groups feeding in larger proportions (HM versus HT groups, χ^2^ = 2.92, df = 1, p = 0.08). Latency to reach the maximum feeding group size observed in a feeding event differed significantly among groups (mean ± SE (minutes), 1^st^ measurement, HM_1_ 17.3 ± 3.8, HM_2_ 20 ± 10, HM_3_ 18.6 ± 3.4, HT_1_ 26.8 ± 4.6, HT_2_ 31.5 ± 6.04; last measurement, HM_1_ 41 ± 14.6, HM_2_ 45 ± 25, HM_3_ 26.7 ± 6.7, HT_1_ 25 ± 3.9, HT_2_ 25.2 ± 4.2; GLM exponential error, Treatment x Time interaction χ^2^ = 9.40, df = 4, n = 499, p = 0.052; Treatment χ^2^ = 30.51, p < 0.0001, df = 4, Time χ^2^ = 5.30, p = 0.021). Contrast analyses showed that these differences were not driven by more related groups reaching maximum feeding group size faster than less related groups (HM versus HT groups, χ^2^ = 3.04, p = 0.08).

### Experiment 3

Relatedness among group members did not affect individual participation in cooperative feeding. The proportion of individuals taking part in feeding events did not differ between sib (S) and non-sib (NS) treatments (S, 0.39 ± 0.04; NS, 0.31 ± 0.04; GLM binomial error, χ^2^ = 2.77, df = 1, n = 28, p = 0.096), and the latency to form feeding groups was similar in both treatments (Wilcoxon test, χ^2^ = 0.0025, df = 1, n = 27, p = 0.96). Within the NS treatment, spiders originating from two different colonies joined feeding groups in equal proportions (GLM binomial error, χ^2^ = 0.13, df = 1, p = 0.71).

## Discussion

We found no indication that social *Stegodyphus dumicola* spiders bias cooperation towards close relatives during feeding. In three different experiments, individuals took part in cooperative feeding events regardless of the level of relatedness of their group members. They aggregated to feed in similar numbers and exploited food with similar feeding efficiency, as shown by comparable rates of food extraction and increase in body weight in the different treatment groups. This suggests lack of nepotism via kin discrimination. Indeed, the finding that individuals participate indiscriminately in feeding events in groups of high and low relatedness suggests overall low discrimination.

In contrast to social *S. dumicola*, kin discrimination has been shown to occur in sub-social species, which are considered a transition stage in the evolution of permanent sociality in spiders^32^,^33^. In sub-social *Stegodyphus*, individuals direct aggression and cannibalism predominantly towards non-sibs^38^, and young in sib groups experience higher foraging efficiency than those in non-sib groups (*S. lineatus*^33^ and *S. tentoriicola*^32^). Similar kin recognition has been shown in other sub-social spider species (*Diaea ergandros*^39^ and *Delena cancerides*^40^). The presumed loss of kin-biased cooperative behaviour in social *S. dumicola* may be attributed to social or ecological contexts: firstly, a high level of relatedness among colony members reduces both the need for kin recognition and the effectiveness of kin-biased cooperation. Secondly, a sedentary foraging mode and life style should reduce the potential for competitive interactions with non-kin and hence the benefit of maintaining a presumed costly recognition system; and thirdly, strong direct benefits of cooperation within the group can outweigh any potential advantages of directing cooperation preferentially towards close kin. Any of these factors could change the cost-benefit ratio of exerting nepotistic behaviour in the transition from sub-social to permanent social behaviour, and result in a reduced role of kin discrimination once permanently sociality had evolved.

### Genetic relatedness within colonies

The breeding system and modes of colony establishment in social spiders result in high genetic homogeneity within colonies. There is no juvenile natal dispersal and spiders mate within the parent colony over multiple generations, resulting in extreme inbreeding^21^,^41^. New nests are established by dispersal of mated females; in *S. dumicola*, single, mated females disperse on silk by ballooning or bridging^42^, and if successful, will produce a clutch of siblings to form the start of a new colony^21^. An alternative mode of colony establishment is by budding or fission of large nests, whereby groups of large juveniles split off the parent nest to form daughter nests that may then become separated from the parent nest^21^. The daughter nests thus contain a subset of the original inbred colony. Both modes of colony establishment result in high relatedness among colony members and the perpetuation of the inbred parental lineage. Movement between colonies is uncommon and restricted to nearby nests^41^, which are likely derived from the same female lineage and therefore composed of genetically similar individuals^34^. Colony establishment by either method involves small breeding populations and this can lead in the long run to overall loss of genetic variation in the population. This process is further enhanced by the female-biased sex ratio and consequent decrease in effective population size. Overall low genetic variation within colonies, across populations and indeed across the entire species’ range characterizes the social spider species that have been investigated to date^21^,^22^,^43^. Given this population genetic structure of inbred colonies with extremely low genetic variation, kin-biased cooperation would seem unlikely to provide any additional benefit to that derived from cooperating indiscriminately within the colony.

### Ecological context

Direct fitness benefits of group living do not require that cooperation be directed preferentially toward kin^7^,^13^. In social spiders, group foraging increases the probability of gaining a successful meal, as prey capture rate increases^44^,^45^ and spiders can subdue and capture larger prey^46^,^47^,^48^. In addition, cooperative foraging reduces per capita energy expenditure on web-construction^44^, and possibly on the production of digestive enzymes. Fitness benefits also come through allomaternal care, as young that are raised cooperatively are larger and have greater survival than those raised solitarily^49^. If the magnitude of these direct benefits increases with increasing group size, processes such as group augmentation may further select for group cohesion^13^,^50^ and override any benefits of kin-biased cooperation. Social spiders have a sedentary foraging mode and lifestyle, they build capture webs surrounding the colony that intercept mobile insect prey, and they undergo the entire life cycle within the colony. Therefore social spiders do not experience competitive interactions with other colonies, which might favour group closure and nest-mate recognition systems as seen in most social insects^20^,^51^,^52^.

Indiscriminate cooperation does not necessarily imply that social spiders lack a colony or nest recognition mechanism. In social insects, for example, nest-mate or kin recognition cues are mediated in part by distinct cuticular hydrocarbon fingerprints^53^, and this may be the case in sub-social spiders as well. Cuticular hydrocarbons were identified as potential kin recognition cues carrying information about family identity in sub-social *S. lineatus*^54^. In social spiders it is frequently noted that unrelated individuals from different colonies, and even heterospecific members of the same genus, are readily accepted into a colony with no obvious aggression^55^. This suggests that social spiders do not respond to cues borne on the spider’s body. However, other sources of information regarding nest or colony identity might be available to spiders, for example silk^56^. Thus, whether colony-related cues occur in social spiders and if so, what might be their possible functions, remain unanswered questions.

### Transition to permanent sociality

Our results are in accordance with a scenario where kin discrimination may have had an important role in the evolution of cooperative group living, particularly in sub-social species that lack the inbred colony structure. At the transition to permanent, inbred sociality, kin-biased cooperation was no longer beneficial, while traits that confer direct benefits were favoured. Kin discrimination and nepotism may be costly traits to maintain; kin discrimination requires sensory capabilities of detection and discrimination among chemical cues, while nepotism would result in fewer foraging opportunities in non-kin environments. To obtain direct benefits of group foraging, individuals only need to cooperate with others in performing a particular task. Direct benefits of cooperation may also be obtained in permanent social groups by means of task differentiation and there is accumulating evidence for task differentiation within social spider colonies^57^,^58^. Task specialization in highly social insects increases group productivity^15^,^59^, and this may be the case in social spiders. In social spiders, greater group productivity results in rapid colony growth and the production of more females that will disperse to establish new colonies^21^,^22^. Collectively, these features suggest that permanent sociality is associated with the emergence of complex social organization where nepotism plays a diminishing role, while direct benefits of task differentiation, cooperative foraging and group defence favours group productivity^50^,^60^.

## Methods

Population genetic studies of social spiders show that they are characterized by strong population genetic structuring due to inbreeding within colonies, metapopulation dynamics and lack of gene flow among local populations^23^,^25^,^34^. Studies of genetic diversity and population structure in social *Stegodyphus* showed that populations are highly differentiated. F_st_ (or phi_st_) estimates are 0.36–0.51 for *S. sarasinorum* in India (geographic distances range between 7 and 1465 km)^25^ and averaged 0.31 for populations of *S. dumicola* in Namibia (geographic distances range between 82 and 515 km)^26^. In order to obtain sufficient genetic differentiation among individuals from different populations we took samples from populations 100–500 km apart.

### Experiment 1: groups of mixed genetic composition

We collected 26 *Stegodyphus dumicola* colonies consisting of juveniles from two populations, (Polokwane and Modjadjiskloof) in South Africa during November 2009. Colonies were transported to Ben-Gurion University of the Negev (Israel) and spiders were kept under laboratory conditions (25°–27 °C, 40% humidity, and 13:11 light: dark cycle), sprayed with water and fed houseflies (*Musca domestica*) and cricket nymphs (*Acheta domestica*) twice a week. To create groups of spiders of homogeneous size, we weighed spiders and formed groups of individuals with similar body mass, creating groups of six spiders in total. All six spiders originated from either the same colony or from two different colonies in a combination of three individuals per colony. Treatments consisted of: (i) sibs from the same colony (S; n = 19); (ii) non-sibs from two colonies from the same population, at most 12 m apart (NS_1_; n = 17); and (iii) non-sibs from two colonies from different populations, (NS_2_; n = 17). Varying the genetic composition of group members allowed us to create decreasing relatedness levels (S > NS_1_ > NS_2_).

### Experiment 2: broods of mixed genetic composition

We collected 50 colonies consisting of late sub-adult spiders from two populations (Seeis and Otavi) in Namibia during January 2010. Colonies were brought to a field laboratory near Otavi, and spiders were raised outdoors under natural conditions, sprayed with water and fed once a week with wild-caught insects (mainly moths, grasshoppers and crickets). Each colony was opened and the sexes separated; males were kept together whereas females were placed inside mesh boxes (12.5 × 12.5 × 10 cm) in groups of four consisting of one reproducing female and three helpers. The reproducing female mated sequentially with two males: a sib from the same colony, a non-sib from a colony of the same population approximately 100 m apart, or a non-sib from a different population approximately 500 km away. Males were presented in alternating order (i.e., one male at a time) and data from reciprocal mating orders were combined in the analysis. By mating females with a different combination of males we produced either ‘homogeneous’ or ‘heterogeneous’ broods. Homogeneous broods shared identical parental genotypes and were sired by a female mated with two of her sibs (HM_1_, n = 13), two non-sibs from different colonies of the same population (HM_2_, n = 8) or two non-sibs from different colonies of different population (HM_3_ = 4). Heterogeneous broods varied in their genetic composition due to different parental genotypes and were sired by a female mated with a sib male and a non-sib male from the same population (HT_1_, n = 36) and a sib and a non-sib from a different population (HT_2_, n = 28). This double-mating design allowed us to control for the number of matings while varying the genetic relatedness structure of the offspring as we can exclude fertilization biases^36^,^37^. In this way, we created broods of decreasing within-group genetic relatedness (HM_1_, HM_2_, HM_3_ > HT_1_, HT_2_). Females of this species do not readily remate, so obtaining double matings required multiple trials, and females that rejected a mating partner were subsequently presented with a different male of the same relatedness level.

Females with egg sacs were transferred to Aarhus University (Denmark) and were kept in laboratory conditions at room temperature and natural photoperiod. Once the eggs hatched and the young had consumed two of the four adult females, each brood was divided into 5 to 7 groups consisting of five spiders with homogeneous body mass (as above).

### Experiment 3: Individual participation

We collected 10 colonies consisting of sub-adult spiders from one population (Otavi) in Namibia during April 2010 and transported them to Aarhus University (Denmark). Colonies were maintained in the laboratory (as above), each inside a transparent plastic terrarium (24.5 × 15 × 15 cm). We marked spiders with non-toxic watercolour dots on the dorsal side of the opisthosoma using an identical colour for individuals from the same colony and different colours for the different colonies. We formed groups of 14–20 individuals to form (i) a control group consisting of siblings originating from the same colony (S, n = 12), and (ii) an experimental group consisting of non-sibs originating from two distant colonies combined in a 1:1 ratio (NS, n = 16).

### Feeding Trials

Spiders were kept in transparent plastic boxes (10 × 5 × 5 cm, Experiments 1 and 2; 17 × 17 × 11 cm, Experiment 3) covered by mesh and containing two crossed wooden sticks to enable web building. Feeding trials started once spiders produced capture webs.

In Experiments 1 and 2 we weighed the prey consisting of either a fly (*Musca domestica* in Experiment 1, *Calliphora sp*. in Experiment 2), or a grasshopper (*Locusta migratoria* in Experiment 1) to the nearest 0.1 mg before introducing it on the capture web. Once the first spider attacked the prey, the number of individuals feeding (i.e. with chelicerae fixed on prey) was scored every 10 minutes during the entire duration of the feeding trial (60 and 120-minute trial, Experiment 1 and 2 respectively). We recorded the maximum feeding-group size (i.e. maximum number of spiders feeding cooperatively) and the duration of time spiders used to reach it. Once the feeding trial terminated, we removed and weighed the prey remains to calculate both the total amount of prey consumed, and the amount per individual. If spiders did not attack the prey within 30 minutes the observation was discarded and the prey was left inside the box for spiders to feed on. For each group we repeated the trials every 10 and 4 days until we obtained 6 and 8 trials in total in Experiment 1 and 2, respectively. Spiders were weighed individually to the nearest 0.1 mg every 14 days to assess growth rate, the change in weight and the variance in weight between treatment groups, over time.

In Experiment 3, we conducted a single feeding trial. We introduced a fly on the web and scored the number of spiders feeding every 5 minutes during a 90-minute trial to record the maximum feeding-group size formation and the proportion of spiders from each colony engaged in communal feeding.

### Statistical analysis

We analysed growth rates (mass was log transformed to meet assumptions of normality of residuals) in Experiments 1 and 2 using mixed model ANOVA, where the interaction between treatment and time tests the prediction that relatedness results in differential growth rates among groups^33^. Feeding efficiency (proportion of mass extracted from prey) was analysed using ANCOVA, and where needed data were transformed to meet assumption of normality of residuals. The maximum proportion of spiders participating in feeding events was analysed with GLM using binomial errors. Latency to reach maximum group-size formation was analysed with GLM using exponential errors. In Experiment 3, to test for the effect of relatedness on individual participation in feeding, we compared the proportion of individuals originating from each of the two colonies that took part in feeding events using binomial tests. The time until the maximum group size gathered was tested using a Wilcoxon test. All statistical analyses were carried out with JMP 10 (SAS Software Inc. Cary, NC, USA).

To estimate the statistical power of our tests we carried out a power analysis on each of the three experiments using G*Power 3.1.9.2. For Experiment 1 and 2 power was calculated for mass increase of groups (growth rate) using the sample size and SD obtained in this study (Experiment 1: N = 46, SD = 4.5; Experiment 2: N = 92, SD = 1.9). The effect size used was estimated using the data in Schneider and Bilde (2008) which tested kin discrimination during foraging in the sub-social spider *Stegodyphus lineatus* using a comparable experimental set up. An effect size of 2.5 mg/group was adapted to a value of 3 mg/group and 2 mg/group for mass increased examined in Experiment 1 and Experiment 2, respectively. For experiment 3 power was calculated for proportion of spiders feeding using the sample size and SD we obtained in this study (n = 16, SD = 0.188). We estimated an effect size based on a single additional spider participating in a foraging event from either colony, corresponding to the smallest possible effect of kinship on foraging participation. We detected a power of 0.67, 0.89 and 0.65 respectively for Experiment 1, Experiment 2 and Experiment 3, at alpha = 0.05. While for each individual experiment there is a risk of 33%, 11% and 35% respectively of not detecting a significant effect (Type II error), we can combine the three power analyses because we have three independent experiments. This gives us a risk of only 1.27% (0.33*0.11*0.35) of a Type II error.

## Additional Information

**How to cite this article**: Berger-Tal, R. *et al.* Evidence for loss of nepotism in the evolution of permanent sociality. *Sci. Rep.* **5**, 13284; doi: 10.1038/srep13284 (2015).

## References

[b1] Hamilton W. D. The genetical evolution of social behaviour. II. J Theor Biol 7, 17–52 (1964). 10.1016/0022-5193(64)90039-6 5875340

[b2] Hamilton W. D. The genetical evolution of social behaviour. I. J Theor Biol 7, 1–16 (1964). 5875341 10.1016/0022-5193(64)90038-4

[b3] Grafen A. Do animals really recognize kin. Animal Behaviour 39, 42–54 (1990).

[b4] Sherman P. W., Reeve H. K. & Pfennig D. W. in Behavioural ecology: an evolutionary approach (eds Krebs J. R. & Davies N. B. ) 69–96 (Blackwell Scientific, 1997).

[b5] Griffin A. S. & West S. A. Kin discrimination and the benefit of helping in cooperatively breeding vertebrates. Science 302, 634–636, 10.1126/science.1089402 (2003). 14576431

[b6] Hepper P. G. Kin recognition . (Cambridge University Press, 2005).

[b7] Clutton-Brock T. Breeding together: Kin selection and mutualism in cooperative vertebrates. Science 296, 69–72, 10.1126/Science.296.5565.69 (2002). 11935014

[b8] Keller L. Indiscriminate altruism: unduly nice parents and siblings. Trends Ecol Evol 12, 99–103 (1997). 21237992 10.1016/s0169-5347(96)10065-3

[b9] Langergraber K. E., Mitani J. C. & Vigilant L. The limited impact of kinship on cooperation in wild chimpanzees. Proceedings of the National Academy of Sciences 104, 7786–7790 (2007). 10.1073/pnas.0611449104 PMC1876525 17456600

[b10] Waldman B. The ecology of kin recognition. Annual Review of Ecology and Systematics 19, 543–571 (1988).

[b11] West S. A., Griffin A. S. & Gardner A. Evolutionary explanations for cooperation. Curr Biol 17, R661–R672, 10.1016/J.Cub.2007.06.004 (2007). 17714660

[b12] Queller D. C. & Strassmann J. E. The veil of ignorance can favour biological cooperation. Biology Lett 9, 20130365 (2013). 10.1098/rsbl.2013.0365 PMC3871334 24132090

[b13] Kokko H., Johnstone R. A. & Clutton-Brock T. The evolution of cooperative breeding through group augmentation. Proceedings of the Royal Society of London. Series B: Biological Sciences 268, 187–196 (2001). 11209890 10.1098/rspb.2000.1349 PMC1088590

[b14] Bergmüller R., Johnstone R. A., Russell A. F. & Bshary R. Integrating cooperative breeding into theoretical concepts of cooperation. Behavioural Processes 76, 61–72 (2007). 17703898 10.1016/j.beproc.2007.07.001

[b15] Ratnieks F. L. W. & Anderson C. Task partitioning in insect societies. Insect Soc 46, 95–108, 10.1007/s000400050119 (1999). 10561126

[b16] Oster G. F. & Wilson E. O. Caste and ecology in the social insects . (Princeton University Press, 1978). 740003

[b17] Boomsma J. J. Kin selection versus sexual selection: Why the ends do not meet. Curr Biol 17, R673–R683, 10.1016/J.Cub.2007.06.033 (2007). 17714661

[b18] Cornwallis C. K., West S. A. & Griffin A. S. Routes to indirect fitness in cooperatively breeding vertebrates: kin discrimination and limited dispersal. J Evolution Biol 22, 2445–2457, 10.1111/j.1420-9101.2009.01853.x (2009). 19824927

[b19] Perrin N. & Lehmann L. Is sociality driven by the costs of dispersal or the benefits of philopatry? a role for kin-discrimination mechanisms. The American Naturalist 158, 471–483 (2001). 10.1086/323114 18707302

[b20] Gordon D. M. Ants distinguish neighbors from strangers. Oecologia 81, 198–200 (1989). 10.1007/BF00379806 28312538

[b21] Lubin Y. & Bilde T. The evolution of sociality in spiders. Adv Stud Behav 37, 83–145, 10.1016/S0065-3454(07)37003-4 (2007).

[b22] Avilés L. in The evolution of social behavior in insects and arachnids 476–498 (Cambridge University Press, 1997).

[b23] Smith D., van Rijn S., Henschel J., Bilde T. & Lubin Y. Amplified fragment length polymorphism fingerprints support limited gene flow among social spider populations. Biological Journal of the Linnean Society 97, 235–246, 10.1111/j.1095-8312.2009.01194.x (2009).

[b24] Johannesen J., Wickler W., Seibt U. & Moritz R. F. A. Population history in social spiders repeated: colony structure and lineage evolution in *Stegodyphus mimosarum* (Eresidae). Mol Ecol 18, 2812–2818, 10.1111/J.1365-294x.2009.04238.X (2009). 19500247

[b25] Settepani V., Bechsgaard J. & Bilde T. Low genetic diversity and strong but shallow population differentiation suggests genetic homogenization by metapopulation dynamics in a social spider. J Evolution Biol 27, 2850–2855, 10.1111/jeb.12520 (2014). 25348843

[b26] Johannesen J., Lubin Y., Smith D. R., Bilde T. & Schneider J. M. The age and evolution of sociality in *Stegodyphus* spiders: a molecular phylogenetic perspective. P R Soc B 274, 231–237, 10.1098/Rspb.2006.3699 (2007). PMC1685853 17148252

[b27] Bilde T., Lubin Y., Smith D., Schneider J. M. & Maklakov A. A. The transition to social inbred mating systems in spiders: role of inbreeding tolerance in a sub-social predecessor. Evolution 59, 160–174, 10.1554/04-361 (2005). 15792236

[b28] Whitehouse M. E. A. & Lubin Y. The functions of societies and the evolution of group living: spider societies as a test case. Biol Rev 80, 347–361, 10.1017/s1464793104006694 (2005). 16094803

[b29] Whitehouse M. E. A. & Lubin Y. Competitive foraging in the social spider *Stegodyphus dumicola*. Animal Behaviour 58, 677–688, 10.1006/anbe.1999.1168 (1999). 10479384

[b30] Rankin D. J., Bargum K. & Kokko H. The tragedy of the commons in evolutionary biology. Trends Ecol Evol 22, 643–651, 10.1016/J.Tree.2007.07.009 (2007). 17981363

[b31] West S. A., Pen I. & Griffin A. S. Cooperation and competition between relatives. Science 296, 72–75 (2002). 11935015 10.1126/science.1065507

[b32] Ruch J., Heinrich L., Bilde T. & Schneider J. M. Relatedness facilitates cooperation in the sub-social spider, *Stegodyphus tentoriicola*. Bmc Evolutionary Biology 9, 10.1186/1471-2148-9-257 (2009). PMC2774699 19860868

[b33] Schneider J. M. & Bilde T. Benefits of cooperation with genetic kin in a sub-social spider. Proceedings of the National Academy of Sciences of the United States of America 105, 10843–10846, 10.1073/pnas.0804126105 (2008). 18658236 PMC2504791

[b34] Johannesen J., Hennig A., Dommermuth B. & Schneider J. M. Mitochondrial DNA distributions indicate colony propagation by single matri-lineages in the social spider *Stegodyphus dumicola* (Eresidae). Biological Journal of the Linnean Society 76, 591–600, 10.1046/J.1095-8312.2002.00082.X (2002).

[b35] Cornwallis C. K., West S. A., Davis K. E. & Griffin A. S. Promiscuity and the evolutionary transition to complex societies. Nature 466, 969–972 (2010). 20725039 10.1038/nature09335

[b36] Tuni C., Goodacre S., Bechsgaard J. & Bilde T. Moderate Multiple Parentage and Low Genetic Variation Reduces the Potential for Genetic Incompatibility Avoidance Despite High Risk of Inbreeding. Plos One 7, 10.1371/journal.pone.0029636 (2012). PMC3250463 22235316

[b37] Schneider J. M. & Lubin Y. Infanticidal male eresid spiders. Nature 381, 655–656 (1996).

[b38] Bilde T. & Lubin Y. Kin recognition and cannibalism in a sub-social spider. J Evolution Biol 14, 959–966 (2001).

[b39] Evans T. A. Kin recognition in a social spider. Proceedings of the Royal Society of London. Series B: Biological Sciences 266, 287–292, 10.1098/rspb.1999.0635 (1999).

[b40] Yip E., Clarke S. & Rayor L. Aliens among us: nestmate recognition in the social huntsman spider, *Delena cancerides*. Insect Soc 56, 223–231 (2009).

[b41] Lubin Y., Birkhofer K., Berger-Tal R. & Bilde T. Limited male dispersal in a social spider with extreme inbreeding. Biological Journal of the Linnean Society 97, 227–234 (2009).

[b42] Schneider J. M., Roos J., Lubin Y. & Henschel J. R. Dispersal of *Stegodyphus dumicola* (Araneae, Eresidae): They do balloon after all! Journal of Arachnology 29, 114–116 (2001).

[b43] Berger-Tal R., Tuni C., Lubin Y., Smith D. & Bilde T. Fitness consequences of outcrossing in a social spider with an inbreeding mating system. Evolution 68, 343–351 (2013). 24111606 10.1111/evo.12264

[b44] Ward P. I. & Enders M. M. Conflict and Cooperation in the Group Feeding of the Social Spider *Stegodyphus mimosarum*. Behaviour 94, 167–182, 10.2307/4534457 (1985).

[b45] Yip E. C., Powers K. S. & Avilés L. Cooperative capture of large prey solves scaling challenge faced by spider societies. Proceedings of the National Academy of Sciences 105, 11818–11822, 10.1073/pnas.0710603105 (2008). PMC2575263 18689677

[b46] Nentwig W. Social spiders catch larger prey: a study of *Anelosimus eximius* (Araneae: Theridiidae). Behavioral Ecology and Sociobiology 17, 79–85 (1985).

[b47] Pasquet A. & Krafft B. Cooperation and prey capture efficiency in a social spider, *Anelosimus eximius* (Araneae, Theridiidae). Ethology 90, 121–133 (1992).

[b48] Rypstra A. L. Prey capture and feeding efficiency of social and solitary spiders: a comparison. Acta Zoologica Fennicci 190, 339–343 (1990).

[b49] Salomon M. & Lubin Y. Cooperative breeding increases reproductive success in the social spider Stegodyphus dumicola (Araneae, Eresidae). Behavioral Ecology and Sociobiology 61, 1743–1750, 10.1007/s00265-007-0406-2 (2007).

[b50] Bilde T., Coates K. S., Birkhofer K., Bird T., Maklakov A. A. & Lubin Y. Survival benefits selects for group living in a social spider despite reproductive costs. J Evolution Biol 20, 2412–2426 (2007). 10.1111/j.1420-9101.2007.01407.x 17956402

[b51] Helanterä H., Strassmann J. E., Carrillo J. & Queller D. C. Unicolonial ants: where do they come from, what are they and where are they going? Trends Ecol Evol 24, 341–349 (2009). 19328589 10.1016/j.tree.2009.01.013

[b52] Vogel V., Pedersen J. S., d’Ettorre P., Lehmann L. & Keller L. Dynamics and genetic structure of Argentine ant supercolonies in their native range. Evolution 63, 1627–1639 (2009). 19154388 10.1111/j.1558-5646.2009.00628.x

[b53] Van Zweden J. S. & d’Ettorre P. in Insect hydrocarbons: biology, biochemistry and chemical ecology Vol. 11 (eds Gary B. J. & Begneres A. –G. ) 222–243 (Cambridge University Press, 2010).

[b54] Grinsted L., Bilde T. & d’Ettorre P. Cuticular hydrocarbons as potential kin recognition cues in a sub-social spider. Behavioral Ecology 22, 1187–1194 (2011).

[b55] Seibt U. & Wickler W. Interspecific Tolerance in Social *Stegodyphus* Spiders (Eresidae, Araneae). Journal of Arachnology 16, 35–39, 10.2307/3705802 (1988).

[b56] Roland C. Chemical signals bound to the silk in spider communication (Arachnida, Araneae). Journal of Arachnology 11, 309–314 (1983).

[b57] Grinsted L., Pruitt J. N., Settepani V. & Bilde T. Individual personalities shape task differentiation in a social spider. Proceedings of the Royal Society B: Biological Sciences, 280, 10.1098/rspb.2013.1407 (2013). PMC3735259 23902907

[b58] Settepani V., Grinsted L., Granfeldt J., Jensen J. L. & Bilde T. Task specialization in two social spiders, *Stegodyphus sarasinorum* (Eresidae) and *Anelosimus eximius* (Theridiidae). J Evolution Biol 26, 51–62 (2013). 10.1111/jeb.12024 23163349

[b59] Jeanson R., Fewell J. H., Gorelick R. & Bertram S. M. Emergence of increased division of labor as a function of group size. Behavioral Ecology and Sociobiology 62, 289–298 (2007).

[b60] Avilés L. & Tufino P. Colony size and individual fitness in the social spider *Anelosimus eximius*. The American Naturalist 152, 403–418 (1998). 10.1086/286178 18811448

